# Supplementary material for: Hybrid Models and Biological Model Reduction with PyDSTool
Source: PLoS Comput Biol. 2012 Aug 9;8(8):e1002628. doi: 10.1371/journal.pcbi.1002628 (PMC3415397; doi:10.1371/journal.pcbi.1002628)
Supplement: Text S4 — Complete source code for the PyDSTool package (version 0.88.120504). Includes API documentation and help files linking to web pages. This file is identical to the current public release on Sourceforge.net. (ZIP) [file pcbi.1002628.s004.zip › PyDSTool/html/PyDSTool.Interval'-pysrc.html]

xml version="1.0" encoding="ascii"?


PyDSTool.Interval'


| Home | Trees | Indices | Help | PyDSTool |
| --- | --- | --- | --- | --- |

Package PyDSTool ::
Module Interval'

[hide private]

[frames] | no frames]

# Source Code for Module PyDSTool.Interval'

```
  1  """Interval class
 
  2  
 
  3    Robert Clewley, June 2005
 
  4  
 
  5  Interval objects have attributes:
 
  6     name: str  (variable label, which DOES NOT have to be unique)
 
  7     typestr: 'int' or 'float' (immutable) - doesn't handle 'complex' yet
 
  8     type: type
 
  9     _loval (low endpoint val): numeric
 
 10     _hival (hi endpoint val): numeric
 
 11     _abseps: numeric
 
 12     issingleton: boolean
 
 13     _intervalstr: str
 
 14  """ 
 15  
 
 16  # Note: The integer intervals will later be used as the basis for
 
 17  # supporting finitely-sampled real ranges.
 
 18  
 
 19  ## PyDSTool imports
 
 20  from utils import * 
 21  from common import * 
 22  from errors import * 
 23  
 
 24  ## Other imports
 
 25  from numpy import Inf, NaN, isfinite, isinf, isnan, array, sign, linspace, arange 
 26  import re, math 
 27  import copy 
 28  
 
 29  MIN_EXP = -15 
 30  
 
 31  # type identifiers
 
 32  re_number = '([-+]?(\d+(\.\d*)?|\d*\.\d+)([eE][-+]?\d+)?)' 
 33  
 
 34  c=1  # contained const 
 35  n=0  # notcontained const 
 36  u=-1  # uncertain const 
 37  
 
 38  
 
 39  __all__ = ['notcontained', 'contained', 'uncertain', 'Interval',
 
 40             'isinterval', 'issingleton'] 
 41  
 
 42  
 


43 -class IntervalMembership(int):


44      """Numeric Interval membership type.""" 
 45  
 


46 -    def __init__(self, *arg):


47          val = arg[0] 
 48          if val == -1: 
 49              self.valstr = 'uncertain' 
 50          if val == 0: 
 51              self.valstr = 'notcontained' 
 52          if val == 1: 
 53              self.valstr = 'contained' 
 54          if self.__int__() not in [-1, 0, 1]: 
 55              raise ValueError('Invalid value for numeric Interval membership type')

 56  
 


57 -    def __repr__(self):


58          return self.valstr

 59  
 
 60      __str__ = __repr__ 
 61  
 


62 -    def __and__(self, v):


63          """Interval `logical AND` for checking interval containment.
 
 64          e.g. if both endpoints are contained then the whole interval is.""" 
 65          sv = self.__int__() 
 66          ov = v.__int__() 
 67          if sv == n or ov == n: 
 68              return notcontained 
 69          elif sv == u or ov == u: 
 70              return uncertain 
 71          else: # sv == ov == c is only remaining case 
 72              return contained

 73  
 


74 -    def __rand__(self, v):


75          return self.__and__(v)

 76  
 


77 -    def __or__(self, v):


78          """Interval `logical OR` for checking interval intersection.
 
 79          e.g. if at least one endpoint is contained then there is intersection.""" 
 80          sv = self.__int__() 
 81          ov = v.__int__() 
 82          if sv == c or ov == c: 
 83              return contained 
 84          elif sv == u and ov == u: 
 85              return uncertain 
 86          else: 
 87              return notcontained

 88  
 


89 -    def __ror__(self, v):


90          return self.__or__(v)

 91  
 
 92  
 
 93  global notcontained, contained, uncertain 
 94  notcontained = IntervalMembership(False) 
 95  contained = IntervalMembership(True) 
 96  uncertain = IntervalMembership(-1) 
 97  
 
 98  
 
 99  
 


100 -class Interval(object):


101      """Numeric Interval class.
 
102  
 
103      Numeric Interval implementation for integer and float types.
 
104  
 
105      If the interval is not specified fully on initialisation then operations
 
106          on the object are limited to set().
 
107      """ 
108  
 


109 -    def __init__(self, name, intervaltype, intervalspec=None, abseps=None):


110  #        if not isinstance(name, str):
 
111  #            raise PyDSTool_TypeError('Name must be a string')
 
112          self.name = name 
113          try: 
114              self.type = _num_equivtype[intervaltype] 
115          except KeyError: 
116              raise PyDSTool_TypeError('Incorrect type specified for interval') 
117          self.typestr = _num_type2name[self.type] 
118          if compareNumTypes(self.type, _int_types): 
119              abseps = 0 
120              self.isdiscrete = True 
121          else: 
122              if abseps is None: 
123                  abseps = 1e-13 
124              else: 
125                  assert abseps >= 0, "abseps argument must be non-negative" 
126              self.isdiscrete = False 
127          self._abseps = abseps 
128          self.defined = False  # default value 
129          self._maxexp = None  # default value, unused for singletons 
130          if intervalspec is not None: 
131              self.set(intervalspec) 
132          else: 
133              # just declare the name and type
 
134              self._loval = None 
135              self._hival = None

136  
 
137  
 


138 -    def info(self, verboselevel=0):


139          if verboselevel > 0: 
140              # info is defined in utils.py
 
141              info(self.__dict__, "Interval " + self.name,
 
142                   recurseDepthLimit=1+verboselevel) 
143          else: 
144              print self.__repr__()

145  
 
146  
 
147      # Return all the interval's relevant data in a tuple
 


148 -    def __call__(self):


149          if self.defined: 
150              return (self.name, self.type, self.typestr,\
 
151                      (self._loval, self._hival)) 
152          else: 
153              raise PyDSTool_ExistError('Interval undefined')

154  
 
155  
 


156 -    def __eq__(self, other):


157          assert isinstance(other, Interval) 
158          return self.defined == other.defined and \
 
159                 self.type == other.type and \
 
160                 self._loval == other._loval and \
 
161                 self._hival == other._hival and \
 
162                 self._abseps == other._abseps

163  
 
164  
 


165 -    def __ne__(self, other):


166          return not self == other

167  
 


168 -    def __le__(self, other):


169          if isinstance(other, Interval): 
170              raise NotImplementedError 
171          elif isinstance(other, _seq_types): 
172              return [o <= self._loval + self._abseps for o in other] 
173          else: 
174              return other <= self._loval + self._abseps

175  
 


176 -    def __ge__(self, other):


177          if isinstance(other, Interval): 
178              raise NotImplementedError 
179          elif isinstance(other, _seq_types): 
180              return [o >= self._hival - self._abseps for o in other] 
181          else: 
182              return other >= self._hival - self._abseps

183  
 


184 -    def __lt__(self, other):


185          if isinstance(other, Interval): 
186              return other._loval - other._abseps > self._hival + self._abseps 
187          elif isinstance(other, _seq_types): 
188              return [o > self._hival + self._abseps for o in other] 
189          else: 
190              return other > self._hival + self._abseps

191  
 


192 -    def __gt__(self, other):


193          if isinstance(other, Interval): 
194              return self._loval - self._abseps > other._hival + other._abseps 
195          elif isinstance(other, _seq_types): 
196              return [o < self._loval - self._abseps for o in other] 
197          else: 
198              return other < self._loval - self._abseps

199  
 


200 -    def __add__(self, val):


201          c = copy.copy(self) 
202          c.set((self._loval+val,self._hival+val)) 
203          return c

204  
 


205 -    def __radd__(self, val):


206          c = copy.copy(self) 
207          c.set((self._loval+val,self._hival+val)) 
208          return c

209  
 


210 -    def __sub__(self, val):


211          c = copy.copy(self) 
212          c.set((self._loval-val,self._hival-val)) 
213          return c

214  
 


215 -    def __rsub__(self,val):


216          c = copy.copy(self) 
217          # switch endpoint order
 
218          c.set((val-self._hival,val-self._loval)) 
219          return c

220  
 


221 -    def __mul__(self, val):


222          c = copy.copy(self) 
223          c.set((self._loval*val,self._hival*val)) 
224          return c

225  
 


226 -    def __rmul__(self, val):


227          c = copy.copy(self) 
228          c.set((self._loval*val,self._hival*val)) 
229          return c

230  
 


231 -    def __div__(self, val):


232          c = copy.copy(self) 
233          c.set((self._loval/val,self._hival/val)) 
234          return c

235  
 


236 -    def __rdiv__(self,val):


237          c = copy.copy(self) 
238          # switch endpoint order
 
239          if isfinite(self._hival): 
240              if self._hival==0: 
241                  new_lo = sign(val)*Inf 
242              else: 
243                  new_lo = val/self._hival 
244          else: 
245              new_lo = val/self._hival 
246          if isfinite(self._loval): 
247              if self._loval==0: 
248                  new_hi = sign(val)*Inf 
249              else: 
250                  new_hi = val/self._loval 
251          else: 
252              new_hi = val/self._loval 
253          if new_hi < new_lo: 
254              # negative and division changes order
 
255              c.set((new_hi,new_lo)) 
256          else: 
257              c.set((new_lo,new_hi)) 
258          return c

259  
 


260 -    def __neg__(self):


261          c = copy.copy(self) 
262          # switch endpoint order
 
263          c.set((-self._hival,-self._loval)) 
264          return c

265  
 


266 -    def __contains__(self, val):


267          testresult = self.contains(val) 
268          if testresult == notcontained: 
269              return False 
270          elif testresult == contained: 
271              return True 
272          else: 
273              raise PyDSTool_UncertainValueError('cannot determine membership '
 
274                                           '(uncertain)', val)

275  
 


276 -    def contains(self, val):


277          """Report membership of val in the interval,
 
278          returning type IntervalMembership.""" 
279          if isinstance(val, _seq_types): 
280              return [self.contains(v) for v in val] 
281          try: 
282              if not self.defined: 
283                  raise PyDSTool_ExistError('Interval undefined') 
284              if self._maxexp is None and not self.issingleton: 
285                  try: 
286                      loexp = math.log(abs(self._loval), 10) 
287                  except (OverflowError, ValueError): 
288                      loexp = 0 
289                  try: 
290                      hiexp = math.log(abs(self._hival), 10) 
291                  except (OverflowError, ValueError): 
292                      hiexp = 0 
293                  self._maxexp = max(loexp, hiexp) 
294              if isinstance(val, _num_name2equivtypes[self.typestr]): 
295                  compval = val 
296                  if compareNumTypes(self.type, _int_types): 
297                      eps = 0 
298                  else: 
299                      eps = self._abseps 
300              else: 
301                  if isinstance(val, _all_int): 
302                      compval = float(val) 
303                      eps = self._abseps 
304                  elif isinstance(val, _all_float): 
305                      # cannot ever compare a float in an integer interval,
 
306                      # unless int(val) == val or val is not finite
 
307                      if isinf(val): 
308                          compval = val 
309                          eps = 0 
310                      elif int(val) == val: 
311                          compval = int(val) 
312                          eps = 0 
313                      else: 
314                          raise PyDSTool_TypeError('Incorrect type of query value') 
315                  elif not val.issingleton: 
316                      # catches non-intervals or non-singleton intervals
 
317                      if not val.defined: 
318                          raise PyDSTool_ExistError('Input interval undefined') 
319                      if not compareNumTypes(val.type, self.type) and \
 
320                         compareNumTypes(val.type, _all_float): 
321                          # meaningless to ask if float interval is contained in an
 
322                          # integer interval!
 
323                          raise PyDSTool_TypeError('Interval type mismatch') 
324                      if compareNumTypes(val.type, self.type) and \
 
325                         compareNumTypes(self.type, _all_int): 
326                          eps = 0 
327                      else: 
328                          eps = max(self._abseps, val._abseps) 
329                          try: 
330                              minexpallowed = math.ceil(-MIN_EXP - self._maxexp) 
331                          except TypeError: 
332                              # _maxexp is None
 
333                              minexpallowed = Inf 
334                          if eps > 0 and -math.log(eps,10) > minexpallowed: 
335                              eps = math.pow(10,-minexpallowed) 
336                      if isfinite(val._loval) or isfinite(self._loval): 
337                          tempIlo = val._loval >= (self._loval + eps) 
338                      else: 
339                          tempIlo = False 
340                      if isfinite(val._hival) or isfinite(self._hival): 
341                          tempIhi = val._hival <= (self._hival - eps) 
342                      else: 
343                          tempIhi = False 
344                      if tempIlo and tempIhi: 
345                          return contained 
346                      elif eps == 0: 
347                          return notcontained 
348                      else: 
349                          # having already tested for being contained, this is
 
350                          # sufficient for uncertainty
 
351                          if isfinite(val._loval) or isfinite(self._loval): 
352                              tempUlo = val._loval > (self._loval - eps) 
353                              tempElo = val._loval <= (self._loval - eps) 
354                          else: 
355                              tempUlo = val._loval == self._loval 
356                              tempElo = False 
357                          if isfinite(val._hival) or isfinite(self._hival): 
358                              tempUhi = val._hival < (self._hival + eps) 
359                              tempEhi = val._hival >= (self._hival + eps) 
360                          else: 
361                              tempUhi = val._hival == self._hival 
362                              tempEhi = False 
363                          if ((tempUlo and not tempEhi) or (tempUhi and \
 
364                                                           not tempElo)) \
 
365                                           and not self.isdiscrete: 
366                              return uncertain 
367                          else: 
368                              # else must be notcontained
 
369                              return notcontained 
370                  else: 
371                      # val is a singleton interval type
 
372                      # issingleton == True implies interval is defined
 
373                      # Now go through same sequence of comparisons
 
374                      if compareNumTypes(val.type, self.type): 
375                          compval = val.get() 
376                          if compareNumTypes(self.type, _all_int): 
377                              eps = 0 
378                          else: 
379                              eps = max(self._abseps, val._abseps) 
380                              try: 
381                                  loexp = math.log(abs(self._loval), 10) 
382                              except (OverflowError, ValueError): 
383                                  loexp = 0 
384                              try: 
385                                  hiexp = math.log(abs(self._hival), 10) 
386                              except (OverflowError, ValueError): 
387                                  hiexp = 0 
388                              minexpallowed = math.ceil(-MIN_EXP - max(loexp,
 
389                                                                       hiexp)) 
390                              if eps > 0 and -math.log(eps,10) > minexpallowed: 
391                                  eps = math.pow(10,-minexpallowed) 
392                      else: 
393                          if compareNumTypes(val.type, _all_int): 
394                              compval = val.get() 
395                              eps = self._abseps 
396                          elif compareNumTypes(val.type, _all_float): 
397                              # cannot ever compare a float in an integer interval
 
398                              # unless bd values are equal to their int() versions
 
399                              if int(val._loval) == val._loval and \
 
400                                 int(val._hival) == val._hival: 
401                                  compval = (int(val[0]), int(val[1])) 
402                                  eps = 0 
403                              else: 
404                                  raise PyDSTool_TypeError('Invalid numeric type '
 
405                                                           'of query value') 
406                          else:  # unexpected (internal) error 
407                              raise PyDSTool_TypeError('Invalid numeric type of '
 
408                                                       'query value') 
409          except AttributeError: 
410              raise PyDSTool_TypeError('Expected a numeric type or a singleton '
 
411                                   'interval. Got type '+str(type(val))) 
412          else: 
413              tempIlo = compval >= (self._loval + eps) 
414              tempIhi = compval <= (self._hival - eps) 
415              if tempIlo and tempIhi: 
416                  return contained 
417              elif eps == 0: # only other possibility (no uncertainty) 
418                  return notcontained 
419              else: 
420                  # having already tested for being contained, this is
 
421                  # sufficient for uncertainty
 
422                  tempUlo = compval > (self._loval - eps) 
423                  tempUhi = compval < (self._hival + eps) 
424                  tempElo = compval <= (self._loval - eps) 
425                  tempEhi = compval >= (self._hival + eps) 
426                  if ((tempUlo and not tempEhi) or (tempUhi and not tempElo)) and \
 
427                               not self.isdiscrete: 
428                      return uncertain 
429                  # else must be notcontained
 
430                  else: 
431                      return notcontained

432  
 
433  
 


434 -    def intersect(self, other):


435          if not isinstance(other, Interval): 
436              raise PyDSTool_TypeError("Can only intersect with other Interval "
 
437                                       "types") 
438          result = None    # default, initial value if no intersection 
439          if self.type != other.type: 
440              raise PyDSTool_TypeError("Can only intersect with other Intervals "
 
441                                       "having same numeric type") 
442          if compareNumTypes(self.type, _all_complex) or \
 
443             compareNumTypes(other.type, _all_complex): 
444              raise TypeError("Complex intervals not supported") 
445          if self.contains(other): 
446              result = other 
447          elif other.contains(self): 
448              result = self 
449          else: 
450              # no total containment possible
 
451              if other.contains(self._hival) is contained: 
452                  # then also self.contains(other._loval)
 
453                  result = Interval('__result__', self.type, [other._loval,
 
454                                                      self._hival]) 
455              elif other.contains(self._loval) is contained: 
456                  # then also self.contains(other._hival)
 
457                  result = Interval('__result__', self.type, [self._loval,
 
458                                                      other._hival]) 
459          return result

460  
 
461  
 


462 -    def atEndPoint(self, val, bdcode):


463          """val, bdcode -> Bool
 
464  
 
465          Determines whether val is at the endpoint specified by bdcode,
 
466          to the precision of the interval's _abseps tolerance.
 
467          bdcode can be one of 'lo', 'low', 0, 'hi', 'high', 1""" 
468  
 
469          assert self.defined, 'Interval undefined' 
470          assert isinstance(val, (_int_types, _float_types)), \
 
471                 'Invalid value type' 
472          assert isfinite(val), "Can only test finite argument values" 
473          if bdcode in ['lo', 'low', 0]: 
474              if self.isdiscrete: 
475                  return val == self._loval 
476              else: 
477                  return abs(val - self._loval) < self._abseps 
478          elif bdcode in ['hi', 'high', 1]: 
479              if self.isdiscrete: 
480                  return val == self._hival 
481              else: 
482                  return abs(val - self._hival) < self._abseps 
483          else: 
484              raise ValueError('Invalid boundary spec code')

485  
 


486 -    def sample(self, dt, strict=False, avoidendpoints=False):


487          """Sample the interval, returning a list.
 
488  
 
489      Arguments:
 
490  
 
491      dt : sample step
 
492  
 
493      strict : (Boolean) This option forces dt to be used throughout the interval,
 
494        with a final step of < dt if not commensurate. Default of False
 
495        is used for auto-selection of sample rate to fit interval
 
496        (choice based on dt argument).
 
497  
 
498      avoidendpoints : (Boolean, default False). When True, ensures that the first and
 
499        last independent variable ("t") values are not included, offset by
 
500        an amount given by self._abseps (the endpoint tolerance).
 
501      """ 
502          assert self.defined 
503          intervalsize = self._hival - self._loval 
504          assert isfinite(intervalsize), "Interval must be finite" 
505          if dt > intervalsize: 
506              print "Interval size = %f, dt = %f"%(intervalsize, dt) 
507              raise ValueError('dt must be smaller than size of interval') 
508          if dt <= 0: 
509              raise ValueError('Must pass dt >= 0') 
510          if compareNumTypes(self.type, _all_float): 
511              if strict: 
512                  # moved int() to samplist's xrange
 
513                  samplelist = list(arange(self._loval, self._hival, dt,
 
514                                           dtype=float)) 
515                  if self._hival not in samplelist: 
516                      samplelist.append(self._hival) 
517              else: # choose automatically 
518                  n = max(round(intervalsize/dt),2) 
519                  dt = intervalsize/n 
520                  samplelist = list(linspace(self._loval, self._hival, n)) 
521              if avoidendpoints: 
522                  samplelist[-1] = self._hival - 1.1*self._abseps 
523                  samplelist[0] = self._loval + 1.1*self._abseps 
524          elif compareNumTypes(self.type, _all_int): 
525              if not isinstance(dt, _int_types): 
526                  raise ValueError("dt must be an integer for integer "
 
527                                          "intervals") 
528              if strict: 
529                  print "Warning: 'strict' option is invalid for integer " + \
 
530                        "interval types" 
531              if avoidendpoints: 
532                  loval = self._loval+1 
533                  hival = self._hival-1 
534                  assert loval <= hival, ('There are no points to return with '
 
535                                          'these options!') 
536              else: 
537                  loval = self._loval 
538                  hival = self._hival 
539              samplelist = range(loval, hival+1, dt) 
540              # extra +1 on hival because of python range() policy!
 
541          else: 
542              raise TypeError("Unsupported value type") 
543          # return a list (not an array) so that pop method is available to VODE Generator, etc.
 
544          return samplelist

545  
 
546      # deprecated syntax
 
547      uniformSample = sample 
548  
 
549  
 


550 -    def set(self, arg):


551          """Define interval in an Interval object""" 
552          if isinstance(arg, _seq_types) and len(arg)==2: 
553              if arg[0] == arg[1]: 
554                  # attempt to treat as singleton
 
555                  self.set(arg[0]) 
556              else: 
557                  self.issingleton = False 
558                  loval = arg[0] 
559                  hival = arg[1] 
560                  #assert not isnan(loval) and not isnan(hival), \
 
561                  #       "Cannot specify NaN as interval endpoint"
 
562                  if not loval < hival: 
563                      print "set() was passed loval = ", loval, \
 
564                            " and hival = ", hival 
565                      raise PyDSTool_ValueError('Interval endpoints must be '
 
566                                      'given in order of increasing size') 
567                  self._intervalstr = '['+str(loval)+',' \
 
568                                      +str(hival)+']' 
569                  if compareNumTypes(type(loval), self.type): 
570                      self._loval = loval 
571                  elif compareNumTypes(self.type, _float_types): 
572                      self._loval = float(loval) 
573                  elif isinf(loval): 
574                      # allow Inf to be used for integer types
 
575                      self._loval = loval 
576                  else: 
577                      raise TypeError("Invalid interval endpoint type") 
578                  if compareNumTypes(type(hival), self.type): 
579                      self._hival = hival 
580                  elif compareNumTypes(self.type, _float_types): 
581                      self._hival = float(hival) 
582                  elif isinf(hival): 
583                      # allow Inf to be used for integer types
 
584                      self._hival = hival 
585                  else: 
586                      raise TypeError("Invalid interval endpoint type") 
587                  self.defined = True 
588          elif isinstance(arg, (_int_types, _float_types)): 
589              assert isfinite(arg), \
 
590                     "Singleton interval domain value must be finite" 
591              if self.isdiscrete: 
592                  # int types or floats=ints only
 
593                  if not int(arg)==arg: 
594                      raise TypeError("Invalid interval singleton type") 
595              else: 
596                  arg = float(arg) 
597              self.issingleton = True 
598              self._intervalstr = str(arg) 
599              self._loval = arg 
600              self._hival = arg 
601              self.defined = True 
602          else: 
603              print "Error in argument: ", arg, "of type", type(arg) 
604              raise PyDSTool_TypeError('Interval spec must be a numeric or '
 
605                                       'a length-2 sequence type')

606  
 


607 -    def __setitem__(self, ix, val):


608          if ix == 0: 
609              self.set((val, self._hival)) 
610          elif ix == 1: 
611              self.set((self._loval, val)) 
612          else: 
613              raise PyDSTool_TypeError('Invalid endpoint')

614  
 


615 -    def __getitem__(self, ix):


616          if self.defined: 
617              if ix == 0: 
618                  return self._loval 
619              elif ix == 1: 
620                  return self._hival 
621              else: 
622                  raise PyDSTool_ValueError("Invalid endpoint") 
623          else: 
624              raise PyDSTool_ExistError('Interval undefined')

625  
 


626 -    def isfinite(self):


627          if self.defined: 
628              if self.issingleton: 
629                  return isfinite(self._loval) 
630              else: 
631                  return (isfinite(self._loval), isfinite(self._hival)) 
632          else: 
633              raise PyDSTool_ExistError('Interval undefined')

634  
 


635 -    def get(self, ix=None):


636          """Get the interval as a tuple or a number (for singletons),
 
637          or an endpoint if ix is not None""" 
638          if self.defined: 
639              if ix == 0: 
640                  return self._loval 
641              elif ix == 1: 
642                  return self._hival 
643              elif ix is None: 
644                  if self.issingleton: 
645                      return self._loval 
646                  else: 
647                      return [self._loval, self._hival] 
648              else: 
649                  raise PyDSTool_TypeError('Invalid return form specified') 
650          else: 
651              raise PyDSTool_ExistError('Interval undefined')

652  
 
653  
 


654 -    def _infostr(self, verbose=1):


655          """Get info on a known interval definition.""" 
656  
 
657          if verbose > 0: 
658              infostr = "Interval "+self.name+"\n" 
659              if self.defined: 
660                  infostr += '  ' + self.typestr+': '+\
 
661                            self.name+' = '+self._intervalstr+ \
 
662                            ' @ eps = '+str(self._abseps) 
663              else: 
664                  infostr += '  ' + self.typestr+': '+self.name+' @ eps = '+ \
 
665                            str(self._abseps) + " (not fully defined)" 
666          else: 
667              infostr = "Interval "+self.name 
668          return infostr

669  
 
670  
 


671 -    def __repr__(self):


672          return self._infostr(verbose=0)

673  
 
674  
 
675      __str__ = __repr__ 
676  
 
677  
 


678 -    def info(self, verboselevel=1):


679          print self._infostr(verboselevel)

680  
 
681  
 


682 -    def __copy__(self):


683          pickledself = pickle.dumps(self) 
684          return pickle.loads(pickledself)

685  
 
686  
 


687 -    def __getstate__(self):


688          d = copy.copy(self.__dict__) 
689          # remove reference to Cfunc self.type
 
690          d['type'] = None 
691          return d

692  
 
693  
 


694 -    def __setstate__(self, state):


695          self.__dict__.update(state) 
696          # reinstate Cfunc self.type
 
697          self.type = _num_name2type[self.typestr]

698  
 
699  
 
700  #--------------------------------------------------------------------
 
701  
 
702  # Exported utility functions
 
703  
 


704 -def isinterval(obj):


705      """Determines whether the given obj is a Interval object.""" 
706      return isinstance(obj, Interval)

707  
 
708  
 
709  # Check whether an interval is a singleton
 


710 -def issingleton(ni):


711      if ni.defined: 
712          return ni.issingleton 
713      else: 
714          raise PyDSTool_ExistError('Interval undefined')

715
```

  


| Home | Trees | Indices | Help | PyDSTool |
| --- | --- | --- | --- | --- |

|  |  |
| --- | --- |
| Generated by Epydoc 3.0.1 on Fri May 4 15:24:17 2012 | http://epydoc.sourceforge.net |
